# Magnetite nanodiscs as vortex-enhanced MRI contrast agents: a novel approach in medical imaging†

**DOI:** 10.1039/d5na01089f

**Published:** 2026-04-01

**Authors:** Elif Koçar, Giuseppe Ferrauto, Syed Bilal Nizami, Vicente Durán Toro, Uzair Ali, Lorenzo Signorelli, Teresa Giannattasio, Marco Micali, Franziska Wasner, René Stein, Rainer Tietze, Marianna Sorrentino, Alessia Corrado, Chiara Papi, Angelo Scarciglia, Enza Di Gregorio, Nicola Toschi, Danijela Gregurec, Allegra Conti

**Affiliations:** a Friedrich-Alexander University of Erlangen-Nuremberg, Department of Chemistry and Pharmacy Henkestr. 91 91052 Erlangen Germany danijela.gregurec@fau.de; b University of Turin, Molecular Biotechnology Center Via Nizza, 52 10126 Torino Italy enza.digregorio@unito.it; c University of Rome Tor Vergata, Department of Biomedicine and Prevention Via Montpellier 1 00133 Rome Italy; d A. A. Martinos Center for Biomedical Imaging – Harvard Medical School/MGH 149 13th Street 02129 Boston MA USA

## Abstract

Magnetic nanodiscs (MNDs) represent a transformative class of anisotropic magnetic nanoparticles with intrinsic vortex magnetization, enabling multifunctional applications in biomedical imaging and therapy. Here, we demonstrate their potential as dual-mode magnetic resonance (MR) contrast agents, a unique feature which is enabled by the high longitudinal relaxivity (*r*_1_ ≈ 40 mM^−1^ s^−1^) at ultralow magnetic fields (<70 µT) in combination with strong transverse relaxivity (*r*_2_ > 150 mM^−1^ s^−1^) at ultrahigh fields (>7 T). This field-dependent relaxivity profile uniquely positions MNDs as versatile *T*_1_/*T*_2_ agents compatible with emerging low-field MRI platforms and high-resolution clinical systems. *Ex vivo* and *in vivo* assessments confirmed clear anatomical localization and preferential hepatic accumulation, suggesting prolonged circulation times due to surface-mediated immune evasion. These properties highlight MNDs as promising candidates for next-generation theranostics, with tunable magnetic responses, high contrast efficiency, and the ability to synergize imaging and neurostimulation.

## Abstract 2

Magnetic nanodiscs (MNDs) represent a transformative class of anisotropic magnetic nanoparticles with intrinsic vortex magnetization, enabling multifunctional applications in biomedical imaging and therapy.

## Graphical abstract

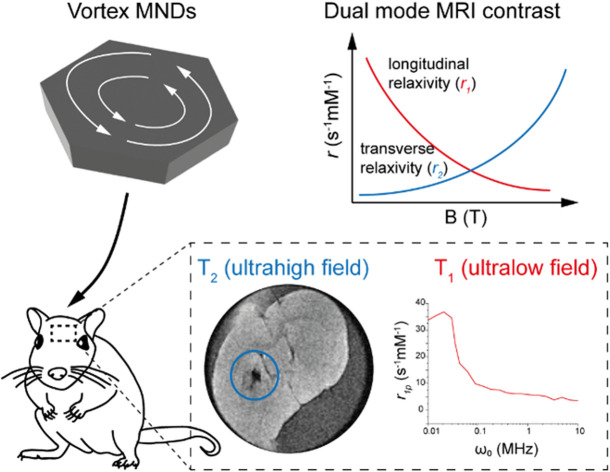

## 1. Introduction

Magnetic resonance imaging (MRI) has been a cornerstone technology in clinical imaging applications and preclinical research for decades, due to its unparalleled ability to provide detailed, high-resolution anatomical, and functional information without ionizing radiation. The underlying mechanism is based on the excitation and relaxation of hydrogen nuclei in water molecules, where tissue-dependent variations in longitudinal (*T*_1_) and transverse (*T*_2_) relaxation times form the basis of imaging contrast. This contrast can be significantly enhanced with *T*_1_ and *T*_2_ contrast agents (CA), which are commonly administered intravenously. These agents accumulate in specific tissues, leading to brighter images in *T*_1_-weighted (*T*_1_*w*) scans or darker images in *T*_2_-weighted (*T*_2_*w*) scans, indicating the biodistribution of the contrast agent administered.^1–4^ The ability to locally enhance tissue contrast depends both on chemical properties, such as magnetic susceptibility and metal ion composition, and physical properties, which include geometry, morphology, crystallinity and hydrodynamic diameter. These parameters influence biodistribution, relaxation efficacy and clinical safety in diagnostic applications.^5^*T*_2_ contrast agents, such as superparamagnetic iron oxide nanoparticles (SPIONs) produce strong localized magnetic fields that accelerate spin–spin relaxation, but often cause distortions in adjacent tissues, a phenomenon known as the blooming effect.^6,7^ This effect can complicate the localization of *T*_2_ agents and the interpretability of hypointense areas, potentially indicative of bleeding, calcification, or metal deposits. Due to these challenges, *T*_1_ contrast agents are preferred in clinical settings. Their positive contrast facilitates the depiction of anatomical details while maintaining high spatial resolution and feasibility to detect various pathological or biological conditions.^8^*T*_1_ agents are predominantly paramagnetic therefore they do not disrupt magnetic field uniformity, ensuring the clarity of surrounding anatomical structures remains intact.^9^ Since the 1980s, manganese ions have been *T*_1_ CA approved by the U.S. Food and Drug Administration (FDA) and the European Medicines Agency (EMA).^10,11^ However, significant safety concerns have emerged.^12^ Gadolinium-based contrast agents (GBCA) are associated with nephrogenic systemic fibrosis (NSF), especially in patients with impaired renal function that lead to prolonged retention of GBCAs and reduced elimination from the body. Additionally, recent studies in individuals with normal renal function have indicated that retention and accumulation of gadolinium in various organs, including the brain, occur after multiple administrations of GBCAs.^13^ Although a causal link with the onset of pathological conditions is under investigation, both the FDA and EMA have issued multiple warnings concerning the risk of long-term heavy metal accumulation in the brain and have restricted the use of linear GBCAs.^14^ This situation highlights the urgent need for the development of more biocompatible and efficient MR-contrast agents.^8,12,15–19^ Superparamagnetic iron oxide nanoparticles (SPIONs) exhibit significantly lower toxicity compared to chemical agents such as GBCA, with numerous studies demonstrating their biocompatibility and minimal cytotoxic effects under physiological conditions.^20^ SPIONs, recognized for their unique magnetic properties, have emerged as promising magnetically active *T*_2_ contrast agents in medical imaging.^21^ These nanoparticles are usually defined as magnetite/maghemite nanoparticles of diameters below 100 nm,^20,22^ and zero net magnetization without an external magnetic field, which minimizes magnetic interactions and reduces particle agglomeration. However, under strong magnetic fields, such as those used in MRI, SPIONs show a strong local magnetic field disturbance relying on superparamagnetic behavior which enhances contrast by shortening relaxation times.^23^ Despite these advantages, SPIONs still face limitations in specificity and detection sensitivity, for example, by creating signal voids that allow the interpretation of only fractions of a voxel. Furthermore, strategies such as advanced polymer surface coatings have been explored to improve their stability, but with limited success in clinical translation.^24,25^

With recent advancements in low-field portable MRI and ultrahigh-field systems for detailed neuroimaging, there is a growing demand for next-generation contrast agents capable of delivering strong, field-tunable relaxivity across a wide range of field strengths.^26,27^ In addition, such agents must offer improved safety, prolonged circulation, and potential for multimodal applications.

In this context, this study presents magnetic nanodiscs (MNDs), a novel class of anisotropic magnetite nanoparticles with vortex alignment of magnetic spins. This unique magnetic property endows them with high saturation magnetisation, while maintaining near-zero magnetization in absence of external magnetic fields. This minimizes aggregation, decreases the demand for contrast agent concentration, and increases colloidal stability. In addition, their low magnetocrystalline anisotropy and exchange stiffness enhance their relaxivity.^28^ Under applied fields, their in-plane magnetic transition enhances both longitudinal and transverse relaxivity, depending on the field strength.

Beyond MRI contrast generation, MNDs were originally developed as nanoscale actuators for remote neuromodulation, where their vortex magnetization enables wireless magnetomechanical stimulation of mechanosensitive ion channels.^28^ This established functional capability distinguishes them from spherical iron oxide nanoparticles and positions MNDs as inherently multimodal materials. In this context, the ability to visualize and quantify their distribution with MRI becomes an essential complementary requirement, particularly for future applications involving localized neural stimulation and monitoring biological responses. Establishing their dual-mode, field-dependent relaxivity is therefore a critical step toward integrating imaging and mechanical actuation within the same nanostructure and toward advancing MNDs as activatable theranostic probes for brain interfaces. We report here a comprehensive characterization of MNDs, from synthesis and magnetic properties to relaxometry, *ex vivo* imaging, and *in vivo* biodistribution, highlighting their potential as field-flexible multimodal magnetic resonance contrast agents for next-generation biomedical imaging and therapy.

## 2. Experimental procedures

### 2.1 Materials

All chemicals used were of analytical grade and were used as received. Sodium acetate, trioctylamine, poly(maleic anhydride-*alt*-1-octadecene) (PMAO), low gelling agar, phosphate buffer salts (3-[4,5-dimethylthiazol-2-yl]-2,5-diphenyl tetrazolium bromide) (MTT) powder, dimethyl sulfoxide (DMSO) and sodium dodecyl sulfate (SDS) were obtained from Sigma Aldrich. Iron(iii) chloride hexahydrate hexane, chloroform, and Tris-acetate-EDTA (TAE) buffer (10×) were obtained from Carl Roth. Oleic acid, phosphate-buffered saline (PBS) (1*×*), GlutaMax™, fetal bovine serum (FBS), Dulbecco's modified Eagle's medium (DMEM), and absolute ethanol were obtained from Fisher Scientific. Ultra-pure (UP) water was produced using a Milli-Q UV water purification system (Millipore).

### 2.2 Synthesis and PMAO coating of magnetic nanodiscs

Magnetite nanodiscs were synthesized following the method of Gregurec *et al.*^28^ Hematite nanodiscs were first prepared by dissolving 0.8 g sodium acetate in 10 mL absolute ethanol and 1 mL of ultrapure water, followed by the addition of 0.273 g FeCl_3_·6H_2_O. The mixture was stirred, sealed in a Teflon-lined autoclave reactor and heated to 180 °C for 18 hours and after cooling down to room temperature washed with UP water and ethanol. Air dried hematite nanodiscs were reduced to magnetite in trioctylamine/oleic acid at 360 °C in 5% H_2_/95% Ar atmosphere, followed by purification with hexane and toluene. To transfer the nanodiscs to the aqueous phase, they were coated with Poly(maleic anhydride-*alt*-1-octadecene) (PMAO, Mw 30 000). A 10 mg mL^−1^ PMAO solution in toluene was mixed with 100 µg of dry nanodiscs and sonicated for one hour. The solvent was evaporated at 150 °C using an oil bath, and the coated nanodiscs were subsequently sonicated in diluted TAE buffer at 80 °C for three hours, washed multiple times with UP water, and stored at 4 °C.

### 2.3 Structural and magnetic characterization of magnetic nanodiscs

Transmission electron microscopy (TEM) for both hematite and magnetite nanodiscs was conducted using a Zeiss EM 912 transmission microscope. Powder X-ray diffraction patterns for both forms were obtained using a Bruker D8 Advance instrument employing Cu Kα radiation (*λ* = 1.5406 Å) over a 2*θ* range of 20° to 80°. Room temperature hysteresis curves, which quantify magnetization saturation and coercivity, were generated using a superconducting quantum interference device (MPMS 3, Quantum Design). The zeta potential and size distribution of the coated magnetite nanodiscs were assessed in UP water (pH 6.8) at room temperature (25 °C) using a Zetasizer Nano ZS device (Malvern Panalytical) dynamic light scattering (DLS).

### 2.4 Evaluation of iron concentration by ICP-MS and relaxometry

The iron content of the studied nanodiscs was quantified both by relaxometry (Stelar SpinaMaster FFC2000, Stelar, Mede, Pavia, Italy) and by ICP-MS (Element-2; Thermo-Finnigan, Rodano (MI), Italy), with results expressed in mol L^−1^. For the preparation of the relaxometry sample, the nanoparticle samples were diluted 1 : 20 (vol/vol) in water and mixed with concentrated HNO_3_ (67%) in a 1 : 9 (vol/vol) ratio, then heated to 160 °C overnight in sealed glass vials to fully dissolve the materials. Post-mineralization, ^1^H-relaxometry analysis was conducted using a Stelar Spinmaster relaxometer (Stelar, Mede, Pavia), employing the inversion recovery (IR) pulse sequence to measure the longitudinal relaxation rate (*R*_1_ = 1/*T*_1_, where *T*_1_ is the water longitudinal relaxation time). Iron concentration was calculated from a standard curve derived from various concentrations of FeCl_3_ mineralized in nitric acid, using the experimental equation:*R*_1_ = *R*_dia_ + [Fe] × *r*_1p_here, *R*_dia_ represents the diamagnetic contribution of nitric acid, set at 0.52 s, and *r*_1p_ is the concentration-normalized iron relaxation rate, at 1.29 mM^−1^ s^−1^.^29^

For ICP-MS sample preparation, nanodisc specimens were mixed with concentrated HNO_3_ (67%) in a 1 : 4 (vol/vol) ratio and subjected to microwave-induced mineralization at 160 °C for 40 minutes using a Milestone MicroSYNTH Microwave Lab Station equipped with optical fiber temperature control and an HPR-1000/6M six-position high-pressure reactor (Bergamo, Italy). A calibration curve was generated using eight standard absorption solutions (Sigma-Aldrich) with iron concentrations ranging from 0.001–0.1 µg mL^−1^.

### 2.5 Characterization of NMR relaxivity

For NMR relaxation characterization, the MNDs were sonicated for 30 minutes at room temperature prior to testing. MND at a concentration of 100 µM were incorporated into low gelling agar at 37 °C, achieving a final concentration of 2% (w/vol). The phantom included a tube filled with agarose gel. The *T*_2_ of the agarose was measured and found to be significantly longer than the *T*_2_ of even the lowest MNDs concentrations. To account for the bulk contribution of the agarose, the *R*_2_ value of the agarose tube was subtracted from the measured *R*_2_ of the samples. This correction ensures that the reported *R*_2_ values accurately reflect the relaxivity of the MNDs, independent of the phantom background. Relaxation rates *R*_1_ and *R*_2_ (= 1/*T*_2_) were measured at *B*_0_ = 0.5 T and 25 °C using a Stelar Spinmaster relaxometer (Stelar, Mede, Italy).

Longitudinal relaxation (*T*_1_) was measured using an Inversion-Recovery (IR) sequence, in which a 180° pulse inverts the longitudinal magnetization, followed by recovery over a range of inversion times (TI) and readout with a 90° pulse.^30,31^ During the recovery period, the magnetization evolves along the main field, so only spin–lattice relaxation contributes to the signal. *T*_2_ does not affect the measurement because no net magnetization is present in the transverse plane prior to readout, allowing *T*_1_ to be accurately extracted by mono-exponential fitting of the recovery curve.

Transverse relaxation (*T*_2_) was measured using a Carr–Purcell–Meiboom–Gill (CPMG) sequence, which isolates spin–spin relaxation by minimizing longitudinal relaxation effects.^32^ In a CPMG experiment, a 90° pulse tips the longitudinal magnetization into the transverse plane, followed by a train of 180° refocusing pulses that generate spin echoes. The decay of echo amplitudes reflects *T*_2_ processes exclusively, as longitudinal recovery occurs on a much longer timescale. Mono-exponential fitting of the echo decay provides an accurate estimate of *T*_2_. Further details on the signal fitting procedures used to determine *T*_1_ and *T*_2_ values at the different magnetic field strengths are provided in the SI.

Full relaxometric profiling of the nanoparticles was achieved *via*^1^H-nuclear magnetic relaxation dispersion (NMRD) profiles, which chart the longitudinal relaxation rate across variable magnetic fields. These profiles were captured on a fast field-cycling Spinmaster relaxometer FFC2000 NMR (Stelar S.n.c., Mede (PV), Italy), spanning magnetic field strengths from 24 µT to 0.47 T (equivalent to 0.0102–20 MHz proton Larmor frequencies). Higher frequency data (20 to 60 MHz) were collected using a Stelar Spinmaster (Stelar, Mede, Pavia) spectrometer coupled with a Stelar VTC-91 temperature controller. Variable-field relaxometry experiments were performed at 25 °C, with temperature stability within ±0.5 °C.

Additional data were obtained using a 7.1 T scanner (Bruker Avance Neo 300 MHz magnetic resonance imaging) and a 14 T Bruker 600 MHz high-resolution NMR spectrometer. MRI measurements at 300 and 600 MHz were conducted at 25 °C, under temperature-controlled conditions.’

Additional information on the NMR relaxivity characterization can be found in the SI.

### 2.6 *In vivo* studies

For this study, male BALB/c mice aged 8 to 10 weeks and weighing approximately 24 ± 3 g were used (Charles River Laboratories, Calco, Italy). The mice were housed under standard conditions with access to standard rodent food and water ad libitum, and they experienced a 12 hour light/dark cycle. All experimental procedures adhered to the Amsterdam Protocol on Animal Protection and complied with both national (D.L.vo 116/92, D.L.vo 26/2014 and subsequent additions) and international laws and policies (2010/63/EU, EEC Council Directive 86/609, OJL 358, December 1987, NIH Guide for the Care and Use of Laboratory Animals, U.S. National Research Council, 1996). The experimental protocol was approved by the Italian Ministry of Health (authorization number 888/2021-PR). For brain extraction, mice were sacrificed by cervical dislocation in accordance with ethical guidelines and their brains were immediately processed for *ex vivo* MRI (see Section 2.7).

### 2.7 MR acquisitions of phantom and *ex vivo* brains

*T*_2_-weighted MRI of MNDs was performed in a custom agarose phantom and in *ex vivo* mouse brain. The phantom contained eight tubes in 1% agarose with MND concentrations ranging from 2.5 to 100 µM and water as a control.

The T_2_-weighted MR imaging acquisitions at 7 T were performed on custom agarose-based phantoms with MND suspensions at varying concentrations to evaluate transverse relaxivity (*r*_2_). Mono-exponential *T*_2_ fitting of multi-slice multi-echo (TE = 3–600 ms, TR = 5000 ms) and additional experiments varying the number of refocusing pulses (RF = 24, 32, 64) were conducted; full details for both are provided in the SI. To characterize the relaxivity and contrast properties of MNDs in brain tissue, and to establish a reference signal profile for their potential presence, we performed experiments by injecting MNDs into *ex vivo* mouse brains. Imaging was conducted at 7 T using a multi-spin-echo sequence (24–64 refocusing pulses). Images were acquired after stereotactic injection of 10 µL MNDs (200 µM [Fe] = 1.04 mM). *T*_2_ maps were generated from MSME data (40 echo times (TE), 10–400 ms; (*x*,*y*,*z*) resolution = (80, 100, 500) µm) by fitting voxel-wise signal decay to mono- and bi-expontential models:Mono: *S*(TE) = *S*_0_*e*^−TE/*T*2^Bi: *S*(TE) = *A*_1_*e*^−TE/*T*2,1^ + *A*_2_*e*^−TE/*T*2,2^

Fitting was performed using MATLAB's Isqcurvefit (Levenberg–Marquardt). Model selection employed AIC and BIC to balance goodness-of-fit and model complexity:AIC = *n* ln(RSS) + 2*k*BIC = *n* ln(RSS) + *k* ln(*n*)where *n* is the number of echos, *k* the number of parameters and RSS the residual sum of squares. The bi-exponential model accounts for heterogeneous relaxation near magnetic particles (fast *T*_2_) and farther away (slow *T*_2_) and was considered superior when ΔAIC or ΔBIC > 10. Binary voxel-wise maps were generated to visualize regions exhibiting dual-component relaxation.

### 2.8 Biocompatibility (MTT and RBC hemolysis assays)

To evaluate the potential for future *in vivo* applications, we evaluated the biocompatibility of PMAO-coated MNDs *in vitro* using the standard MTT assay to determine their impact on cell viability and proliferation. HEK293 cells were cultured in a 96-well plate at a seeding density of 0.01 × 10^6^ cells/well in DMEM supplemented with 10% FBS and 2 mM GlutaMAX™. The following day, cells were treated with different concentrations of PMAO-coated MNDs: 50 µg mL^−1^, 100 µg mL^−1^, 150 µg mL^−1^, and 200 µg mL^−1^, corresponding to [Fe] levels of 0.05%, 0.1%, 0.15%, and 0.2% (w/vol), respectively. The cells were then incubated with MTT reagent, the resultant formazan crystals were dissolved in DMSO, and absorbance was measured at 590 nm using a SpectraMax M2 spectrometer from Molecular Devices.

A hemolysis assay was performed on red blood cells (RBCs) collected from the tail vein of 14–16-week-old male BALB/c mice weighing approximately 25 ± 3 g, using a 27-gauge syringe preloaded with heparin. Blood was diluted in fresh PBS and centrifuged at 2300 rpm for 8 minutes to pellet the cells. The RBCs were washed, recentrifuged, and then exposed to MNDs at concentrations of 0.2 mM or 0.5 mM ([Fe] corresponding to 46 µg mL^−1^ and 116 µg mL^−1^, respectively) for 30 minutes at room temperature. After incubation, the samples were centrifuged, and the supernatant was collected to measure the released hemoglobin spectrophotometrically at 413 nm (Soret's band) using a 6715 UV/Vis spectrophototometer (JEOL). RBCs incubated in fresh PBS served as controls. Lysed red blood cells (using osmotic shock) were used as a reference for fully released Hemoglobin.

### 2.9 *In vivo* biodistribution

Animal experimentation was conducted according to the approved protocol number 888/2021-PR. MNDs were administered intravenously in 10 week wild type balb/c male mice (weight 23 ± 3, *N* = 3). Animals received an intravenous dose of 100 µL per mouse, corresponding to 90 µg Fe per mouse (4 mg Fe kg^−1^), with the MND suspension prepared at 1 mg Fe mL^−1^ (90 µL MND suspension + 10 µL 10× PBS to adjust the osmolarity of the solution. MRI scans were performed using a 7 tesla scanner (Bruker Avance 300 spectrometer equipped with microcoil), with a Rapid Acquisition with Refocused Echoes (RARE) sequence with the following parameters (TR = 4000 ms, TE = 5.5 s, FOV = 1 cm × 1 cm, slice thickness = 1 mm, RARE factor = 32, matrix size 128 × 128). Images were taken at multiple time points: before injection (baseline) and 5 minutes, 1 hour, 4 hours, and 24 hours after injection. The contrast in the scan was analyzed considering the signal variation [(post − pre)/pre) × 100] at the different time points, to assess the biodistribution and temporal dynamics of nanoparticle accumulation in different organs.

### 2.10 ICP-MS

After completion of MRI biodistribution experiments (at 24 h), the animals were sacrificed and organs (liver, kidneys, spleen and muscles) were explanted, weighed, and processed for ICP-MS analysis. Each tissue sample was treated with 1 mL of concentrated nitric acid (HNO_3_, 70%). Following complete dissolution of the tissues, the samples were further digested using microwave heating (MicroSYNTH Microwave Labstation equipped with optical fiber temperature control and an HPR-1000/6M six-position high-pressure reactor, Milestone, Bergamo, Italy). After digestion, the volume of each sample was adjusted to 2 mL using ultrapure water. The solutions were then filtered through a 0.45 µm filter and analyzed by ICP-MS for Fe^3+^ quantification using a Thermo Scientific ELEMENT 2 ICP-MS (Finnigan, Rodano, MI, Italy). Quantification was performed using a calibration curve generated from four iron absorption standard solutions (Sigma-Aldrich) in the range of 0.005–0.1 mg mL^−1^. The total Fe^3+^ mass retained in each specimen was calculated relative to the weight of the tumor tissue (expressed as mg of Fe^3+^ per g of tissue), after subtracting the blank values obtained from untreated control animals (*N* = 3 per group). Iron concentration in each organ was normalized to the corresponding organ in untreated control animals. This approach allowed comparison of organ-specific iron accumulation relative to baseline levels in healthy mice.

## 3. Results and discussion

### 3.1 Synthesis, characterization, and *in vitro* biocompatibility of magnetic nanodiscs

**Fig. 1:**
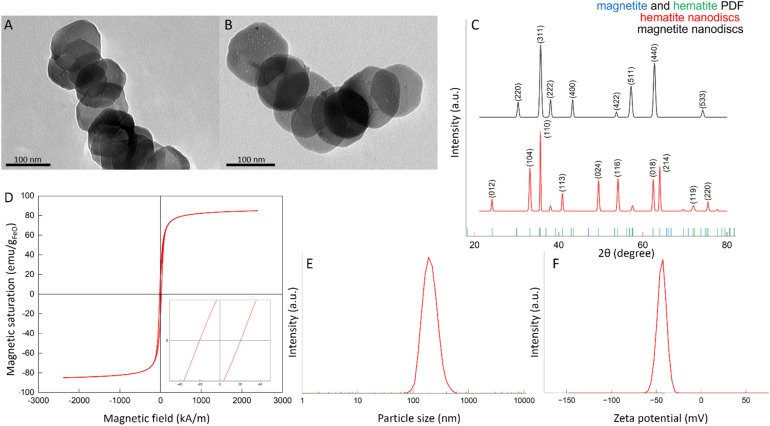
Morphological and magnetic characterization of MNDs. (A) TEM micrograph of hematite and (B) magnetite MNDs of 127 ± 17 nm diameter without polymer coating. (C) XRD spectrum of magnetite (black) and hematite (red) nanodiscs with magnetite and hematite references. (D) Typical magnetite hysteresis showing high saturation magnetization (Ms) and high coercivity (shown in the inset). (E) Determination of the average size by DLS for coated MNDs with PMAO in water. (F) Average zeta potential measurement of MNDs coated with PMAO in water.

The MNDs were synthesized by a two-step method involving hydrothermal synthesis of hematite templates characteristic by hexagonal nonmagnetic lattices, followed by hydrogen-assisted reduction to magnetite phase, as we reported previously.^28^ Transmission electron microscopy (TEM) confirmed preservation of the hexagonal disc morphology after reduction (Fig. 1A and B), with an average diameter of around 127 nm. A detailed size histogram can be found in the SI (Fig. S1). X-ray diffraction (XRD) analysis corroborated the structural transition from nonmagnetic hematite to magnetite (Fig. 1C) with the measured lattice constant (*a* = 8.32 Å) and interplanar spacing (*d* = 2.51 Å) consistent with magnetite's inverse spinel structure.^33–35^

Magnetic characterisation revealed a hysteresis curve (Fig. 1D), high saturation magnetisation (Ms) of approximately 80 emu g^−1^ and coercivity of 36 kA m^−1^, typical of our previously reported MNDs.

The resulting vortex-anisotropic magnetic configuration with high Ms enables minimal net magnetization in the absence of an external field, preventing aggregation and enhancing colloidal stability, crucial properties for biomedical applications.^28^

To ensure water dispersibility and biocompatibility, MNDs were functionalized with poly(maleic anhydride-1-*alt*-octadecene) (PMAO). The resulting size distribution (Fig. 1E, S3) revealed an average hydrodynamic diameter of 201 ± 1 nm. Transmission Electron microscopy (TEM) indicated a core size before the coating of 127 ± 17 nm which was smaller than the value measured by DLS. This discrepancy is attributed to the presence of a hydrated polymer layer and to slight clustering of MNDs in solution, both of which contribute to an increased particle diameter in aqueous solution. These effects also widen the size distribution, explaining the broader SD observed after coating. A polydispersity index (PDI) of 0.17 was observed for the DLS measurements. In general a PDI value between 0.1 and 0.25 indicates a narrow size distribution whereas a PDI greater than 0.5 indicates a broad distribution.^36^ Comparable PDI values have been reported in the literature for iron-based small nanoparticles used as contrast agents in MRI.^37^ These results are aligned with our findings on long term stability of coated MNDs.^38^ The zeta potential revealed a surface charge of −40.7 ± 0.8 mV (Fig. 1F), confirming a successful coating with negatively charged carboxylic groups of hydrolyzed PMAO.^28^ Data are shown as Data are as mean ± SD (DLS: *n* = 3; zeta potential: *n* = 5).

Additionally, the magnetic volume susceptibility of an MNDs was determined as 1.14× 10^−3^ a.u., which aligns with theoretical expectations for vortex-state MNDs.^28^ The key physicochemical parameters are summarized in Table 1.

**Table 1:** Physicochemical Properties of MNDs

Property	Value
Average diameter (by TEM)	127 ± 17 nm
Hydrodynamic diameter (by DLS) in ddH_2_O^a^	201 ± 1 nm
Hydrodynamic diameter (by DLS) in PBS^a^	218 ± 07 nm
*Z*-potential (in dd H_2_O)^a^	−40.7 ± 0.83 mV
*Z*-potential (in PBS)^a^	−22 ± 1.2 mV
Magnetic volume susceptibility^b^	1.14 × 10^−3^ a.u.
Interspacing value *d* (from XRD)	2.51 Å
Lattice constant (from XRD)	8.3247 Å

a PMAO-coated MNDs.

b Theoretical value.

**Fig. 2:**
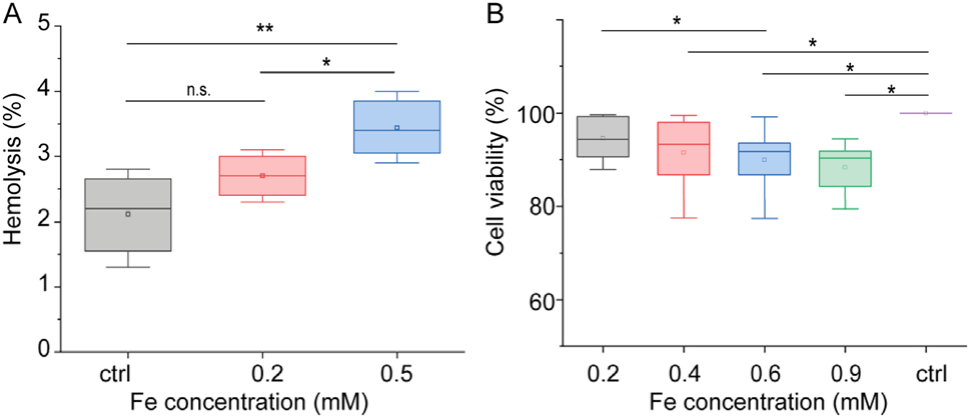
Biocompatibility assays. (A) Hemolysis test. The percentage of hemoglobin released by RBCs after incubation in the presence of MNDs for 30 min at room temperature ([Fe] = 0.2 mM or 0.5 mM). (B) Cellular viability resulting from the MTT assay in HEK293 cells incubated with various concentrations of PMAO coated MNDs and control (no MND). Statistical evaluation was performed using a one-way analysis of variance (ANOVA) and Tukey's comparison of means. *p < 0.5 and **p < 0.01.

To evaluate MNDs biocompatibility *in vitro*, the standard MTT assay revealed concentration-dependent cytotoxicity in HEK293 cells, with cell viability remaining above 75% at concentrations up to 100 µg mL^−1^ (0.4 µM Fe), and moderate reductions at higher doses (Fig. 2B). In parallel, red blood cell (RBC) hemolysis remained below 5% across all tested concentrations (0.2–0.5 mM), indicating minimal hemocompatibility issues (Fig. 2A). These results confirm that PMAO-coated MNDs exhibit low cytotoxicity and high blood compatibility, supporting their suitability for intravenous administration and *in vivo* MRI studies.

### 3.2 Characterization of relaxation by NMR and magnetic resonance imaging

#### 3.2.1 Longitudinal relaxivity

**Fig. 3:**
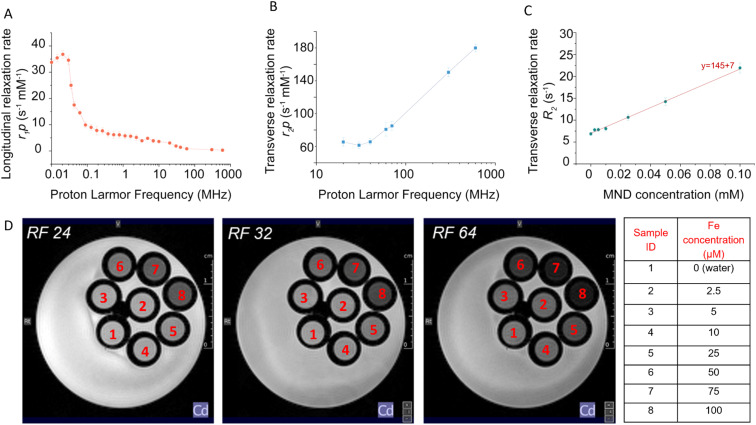
Transverse relaxivity measurements. (A) Longitudinal and (B) transverse relaxivity of PMAO-coated MNDs from 20 to 600 MHz. (C) Linear calibration of the R2 MND concentrations (corresponding to iron concentrations from 0 to 100 µM) to determine the detection limit (LD) at 7 tesla. (D) Phantom magnetic resonance images acquired at 7 T to assess the concentration-dependent *T*_2_ contrast performance of PMAO-coated MNDs at this magnetic field strength.

The asses the longitudinal relaxivity (*r*_1p_) properties of the PMAO-coated MNDs, we recorded a full proton nuclear magnetic relaxation dispersion (^1^H-NMRD) across a wide range of magnetic fields, from 0.23 µT to 1.41 T, supplemented by 7 T (MRI) and 14 T (high-resolution NMR) (Fig. 3A).

The NMRD profile exhibited a distinct peak in longitudinal relaxivity at very low magnetic fields (below 70 µT, corresponding to frequencies under 0.03 MHz), where relaxivity reached values around 40 mM^−1^ s^−1^. This high *r*_1p_ at ultralow field arises from large particle size, its surface to volume ratio, anisotropic geometry and slow water exchange near the MND surface.^39^ This interpretation is further supported by TEM and SEM imaging (Fig. S2), which revealed clusters of MNDs on the order of several hundred nanometres. Such clustering is expected to slow rotational correlation times (*τR*), enhancing low-field *T*_1_ relaxivity by promoting outer-sphere relaxation mechanisms.^40–42^ At intermediate frequencies (100–400 kHz), *r*_1p_ values decreased to 15–20 mM^−1^ s^−1^ while in the clinical MRI range (1–7 T, 40–300 MHz), the *r*_1p_ values decreased further, confirming the field-dependent nature of the performance of *T*_1_. These findings support the application of MNDs and *T*_1_ contrast agents in low field MRI, including portable or emerging low field MRI technologies.^26,27^ The observed NMRD profile is also consistent with previous reports on hyperthermia-efficient anisotropic, shape-anisotropic magnetic structures.^43,44^

#### 3.2.2 Relaxivity comparison with GBCAs

GBCAs are commonly used in clinical MRI due to their *T*_1_ relaxivity values of approximately 3–5 mM^−1^ s^−1^ at conventional field strengths (1.5–3 T).^45^ However, at ultralow magnetic fields, below mT range *r*_1_ relaxivity of GBCAs increases significantly, often approaching or exceeding 10 mM^−1^ s^−1^ depending on molecular properties and environment.^46^

In this ultralow-field regime, longer rotational correlation times and slower molecular tumbling enhance contrast agent efficacy by increasing inner-sphere proton exchange efficiency.^47^ Notably, MNDs show a peak longitudinal relaxivity (*r*_1_) of approximately 40 mM^−1^ s^−1^ at magnetic fields below 70 µT, which exceeds the relaxivity values reported for several commonly used GBCAs under similar conditions. This highlights their potential suitability as *T*_1_ contrast agents for emerging ultralow-field MRI applications.

Table 2 summarizes the *r*_1_ values of different GB-CAs measured at magnetic field strengths below 70 µT. All values were obtained at room temperature. For agents available in multiple molecular sizes, the highest reported relaxivity values are included, as *r*_1_ tends to increase with increasing molecular weight.^48,49^

**Table 2:** MRI Relaxivity of Gadolinium-Based Contrast Agents (GB-CAs) at Different Magnetic Field Strengths. *r*_1_ values are expressed in (mM^−1^ s^−1^)

GB compound	*r*_1_ (ULF)^a^	Biocompatible	Ref.
Gd-DTPA	12	Y	50 and 51
Gd-DOTA	12.5	Y	50 and 52
Gd-1,7-DOTAGA2	12	Y	51 and 53
Gd-HTTAHA	14	n/a	50
Gd-DTPA-BC	12.5	Y	54
Gd(AAZTA-C2-COOH)	14	n/a	55
Gd(AAZTA-C4-COOH)	16	n/a	56

a ULF – ultralow field (<0.1 T).

As shown in the Table 2, some of the listed compounds are not biocompatible or lack sufficient biocompatibility data, whereas our MNDs have demonstrated good biocompatibility, supporting their potential use for *in vivo* applications.

#### 3.2.3 Transverse relaxivity

The transverse relaxivity (*r*_2p_) of the PMAO-coated MNDs was evaluated across magnetic field strengths ranging from 0.5 mT to 14 T (Fig. 3B). The *r*_2p_ values increased with frequency and magnetic fields, reaching 150 s^−1^ mM^−1^ at 7 T, and up to 180 s^−1^ mM^−1^ at 14 T. MNDs likely contribute to the field-dependent relaxometric behavior by altering the diffusion of water and improving local magnetic susceptibility, thus supporting the observed high transverse relaxivity at high magnetic fields. This aggregation-induced effect is consistent with previous findings showing that nanoparticle clustering can amplify transverse relaxation by creating stronger local magnetic field inhomogeneities.^57^ This behavior is particularly relevant at high field strengths, where susceptibility-driven dephasing becomes a dominant mechanism that influences *r*_2_ values.^58^

To visualise contrast efficiency, we imaged agarose-based phantoms containing MNDs at various concentrations (2.5–100 µM) using a 7 T MRI system. Fig. 3D shows representative *T*_2_-weighted MRI images acquired at 7 tesla using varying *T*_2_-weighting levels, where the RARE factor (RF) angle was varied; ((a) RF = 24°, (b) RF = 32°, and (c) RF = 64°). The phantom consisted of tubes filled with varying concentrations of MNDs (100, 75, 50, 25, 10, 5, and 2.5 µM) and a water control. As the RF value increased, the contrast of *T*_2_ became more pronounced due to the longer duration of the echo train, highlighting differences in transverse relaxation. This effect is especially visible at higher MND concentrations, where the dephasing because of magnetic susceptibility is stronger. A clear concentration-dependent signal drop was observed, with visible contrast at concentrations as low as 10 µM (Fig. 3D). The images demonstrate the clear dependence of *T*_2_ signal attenuation on both MND concentration and the degree of *T*_2_-weighting, reinforcing their role as effective *T*_2_ contrast agent at ultra high magnetic fields.

A distinct negative contrast was visible, particularly at lower concentrations of MNDs (*e.g.* tube #5, [MND] = 0.025 mM).

From the linear calibration of the concentration of R2 MND (*y* = 145*x* + 7) we evaluated the limit of detection (LOD) based on the standard 3-sigma criterion. The LOD, defined as:
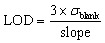
was calculated using the standard deviation (*σ*_blank_) of the water-only (0 mM) control signal (*σ*_blank_ = 0.5 s^−1^). Substituting into the formula:
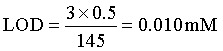


Fig. 3C shows the *R*_2_*versus* concentration curve, which was derived from the *T*_2_ map of a phantom containing different Iron concentrations. The *T*_2_ map was generated using an MSME sequence acquired at 7 tesla with variable echo times and is included in the SI (see Fig. S4 and SI for further details about the MRI acquisition). Using these values against the concentrations of the MNDs in each tube, a relaxivity of 147 mM^−1^ s^−1^ was determined at 7 tesla. To highlight differences in nanoparticle concentration, images acquired at different RFs are also shown. In Fig. 3D, representative *T*_2_-weighted MR images (RARE sequences) illustrate how signal darkening correlates with the presence and concentration of MNDs.^59–61^

The relaxivity profiles suggest the use of the utility of MNDs as a *T*_1_ agent in ultra low-field MR acquisition, while their performance in ultrahigh magnetic field regimes (>3 T) endows them with applicability as a *T*_2_ contrast agent.

To characterize the nature of the MNDs as MRI contrast agents, we evaluated the ratio of transverse to longitudinal relaxivity (*r*_2_/*r*_1_) at the different field strengths. This ratio is widely used to classify contrast agents: low *r*_2_/*r*_1_ values (typically < 5) indicate *T*_1_-dominant behavior, high values (*e.g.*, > 10) reflect predominant *T*_2_ contrast, and intermediate values may suggest potential for dual-mode contrast.^62^ As can be seen in Fig. S5, in our dataset, the *r*_2_/*r*_1_ ratios range from approximately 25 to 650 across the investigated field strengths (10–600 MHz), with *r*_2_ markedly greater than *r*_1_ at all frequencies. Based on these high *r*_2_/*r*_1_ values, the MNDs exhibit strong transverse relaxivity dominance and behave predominantly as *T*_2_ contrast agents. No field range exhibited *r*_2_/*r*_1_ values sufficiently low to indicate significant *T*_1_ predominance or true dual-mode behavior.

#### 3.2.4 Relaxivity comparison with other iron oxide nanostructures

To contextualize the dual-mode relaxivity profile of MNDs, we compared their field-dependent *r*_1_ and *r*_2_ relaxivities with those reported for other iron oxide nanostructures including spherical SPIONs, nanocubes, and nanoclusters (Table 3). While many iron oxide systems exhibit some degree of field-dependent relaxivity, the combination of ultralow-field *r*_1_ = 40 mM^−1^ s^−1^ and high-field *r*_2_ > 150 mM^−1^ s^−1^ observed in MNDs is notably distinct. Although MNDs were not synthesized explicitly for optimizing MRI contrast, their vortex state and anisotropic geometry impart relaxometric behavior not observed in spherical iron oxide particles, including enhanced low-field *r*_1_ and strong high-field *r*_2_ responses. This property profile is distinct from classical SPION-based agents and constitutes a novel relaxometric signature for this nanostructure. Spherical and cubic iron oxide nanoparticles typically demonstrate strong *T*_2_ relaxivity at high fields but lack comparable *T*_1_ performance at ultralow fields.^8,42,63–65^ Among the studied systems (Table 2), the plate geometry indeed plays a role in increasing both longitudinal and transverse relaxometry properties of iron oxide nanoparticles, as demonstrated by Zhou *et al.*^64^ In agreement with these findings, here observed superior *r*_1_ of MNDs at <0.1 T suggests a mechanism beyond general field dependence, likely related to their large size, vortex magnetization, and anisotropic disc geometry.

**Table 3:** MRI Relaxivity of Iron Oxide Nanostructures at Different Field Strengths. All *r*_1_ and *r*_2_ values are expressed in (mM^−1^ s^−1^)

Nanoparticle	Size	*r*_1_ (ULF)^a^	*r*_1_ [mM^−1^ s^−1^]/IF^b^	*r*_1_ [mM^−1^ s^−1^]/HF^c^	*r*_2_ [mM^−1^ s^−1^]/ULF^a^	*r*_2_ [mM^−1^ s^−1^]/IF^b^	*r*_2_ [mM^−1^ s^−1^]/HF^c^	Ref.
SPIONs (spheres)	10 nm	n/a	n/a	n/a		25/0.47 T	50/7 T	8
Nanocubes	20 nm and 100 nm assemblies	n/a	4–5/1.41 T	5/1.41 T	n/a	680/1.41 T	n/a	63
Nanoclusters (SPIONs)	Aggregates of 5–10 nm	n/a	n/a	6–8/3–10 T	n/a		30–70/5–10 T	42
MNDs (this study)	127 nm vortex nanodiscs	40/<70 µT	10/0.5–1.5 T	3/3 T	n/a	30/3 T	150/7 T	This study
Nanoplates	3–9 nm	n/a	11–40/0.5 T	n/a	n/a	70–300/0.5 T	n/a	64
SPIONs (ferumoxytol)	17–30 nm	36.8/64 mT	19/3 T	n/a		10–87/0.6–3 T	n/a	65

a ULF – ultralow field (<0.1 T).

b IF – intermediate field (0.5–1.5 T, clinical MRI).

c HF – high field (>3 T (including 7 T or 14 T)).

Despite not being designed as conventional iron oxide-based contrast agents, our MNDs nonetheless exhibit relaxivity properties while their primary function is designed for neuromodulation. MND size and morphology were carefully optimized to minimize toxicity, while their composition (magnetite) and physical characteristics (∼100 nm hydrodynamic diameter) naturally account for the observed dual relaxivity. At ultralow magnetic fields, the large magnetic moment and slower rotational dynamics enhance longitudinal relaxation (*r*_1_), whereas at higher fields, clustering and magnetic moment increase local susceptibility, yielding dominant transverse relaxivity (*r*_2_). Consequently, the distinct *r*_1_ at low fields and *r*_2_ at high fields arise from the intrinsic properties of magnetite in combination with particle size and clustering, rather than from deliberate strategies to tune relaxivity.

These findings support the assertion that MNDs are not merely field-tuned iron oxide particles but possess structural and magnetic features that uniquely enable dual-mode MRI contrast.

### 3.3 *Ex vivo* MR imaging with MNDs

**Fig. 4:**
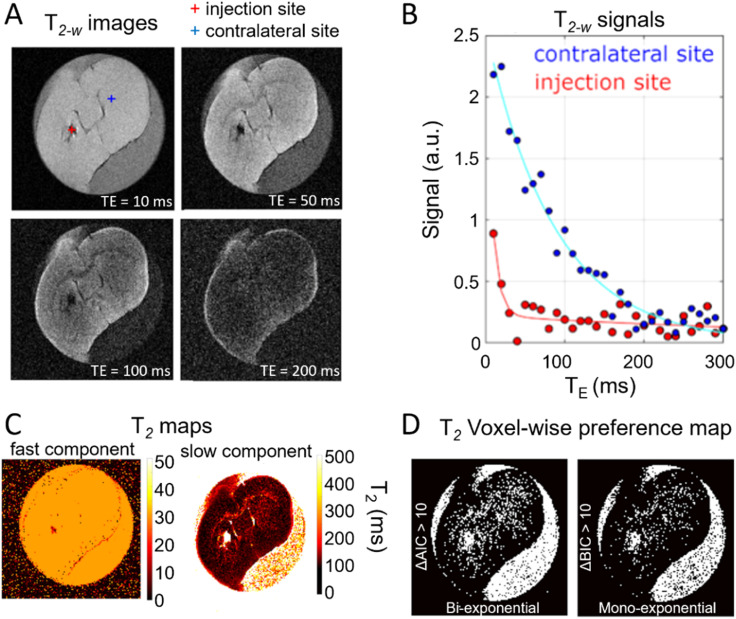
*Ex vivo* magnetic resonance characterization of MNDs in brain tissue. (A) *T*_2_-weighted MR images acquired at 7 tesla, at multiple echo times show hypointense signals at the injection site (injection of 10 µL MNDs, site highlighted in red), indicating accumulation of MND. The contralateral region (blue) served as a control and did not show no signal change. (B) Signal decay curves from representative voxels in the injection and contralateral regions, fitted with a bi-exponential model. Faster transverse relaxation is evident in the injection site due to local MND-induced susceptibility effects. (C) Parametric *T*_2_ maps derived from the bi-exponential fit, separating the fast-relaxing component (proximal to MNDs) from the slower bulk-like component. (D) Binary AIC/BIC maps identify voxels (white) where the bi-exponential model significantly outperforms the mono-exponential model (AIC/BIC > 10), confirming two distinct proton populations. These results underscore the relevance of bi-exponential modeling in tissues labeled with ultra-high relaxivity MND, enabling enhanced detection and interpretation of MND-tissue interactions.

Fig. 4 summarizes the *ex vivo* MR characterization of MNDs. *T*_2_-weighted magnetic resonance images of a representative brain section are shown in Fig. 4A for different echo times. As a demonstration of their effectiveness as MRI contrast agents, MNDs appear as localized hypointense areas (dark spots) in the images. The injection site is colored red in the upper left panel, while the contralateral brain region, colored blue, served as a control and did not show significant changes in signal with respect to surrounding tissue. Fig. 4B presents the signal decay curves extracted from representative voxels in the injection site and the contralateral region. These decays were fitted using a bi-exponential model, and the resulting fits are shown as blue and red curves for the contralateral and injection sites, respectively. A markedly faster transverse decay is observed in the injection region, consistent with the local accumulation of MNDs, which strongly affect the transversal relaxation of protons. Fig. 4C displays the corresponding parametric *T*_2_ maps, separately highlighting the fast-relaxing component, associated with water protons in close proximity to MNDs, and the slower component, representative of bulk-like water. These maps confirm the spatial localization and relaxation impact of the MNDs. Finally, Fig. 4D shows binary maps where both the Akaike and Bayesian Information Criterion (AIC/BIC) values exceed a threshold of 10, indicating that a bi-exponential model provides a significantly better fit in the injection site (highlighted in white). This supports the presence of two distinct populations of water protons, consistent with the heterogeneous microenvironment induced by the MNDs. These results highlight the utility of the bi-exponential relaxation model in interpreting *T*_2_-weighted MR signals in tissues labelled with MNDs exhibiting ultra-high transverse relaxivity. The separation of signal components allows for more precise characterization of nanoparticle–tissue interactions and provides a robust framework for evaluating advanced MRI contrast agents. MNDs were administered at a dose of 2.4 mg Fe kg^−1^, which is somewhat lower compared to clinically used SPIONs such as ferumoxytol, typically administered at 3–7.5 mg Fe kg^−1^.^66^ This demonstrates the utility of the MNDs in clinical settings with the benefit of efficient low-field *T*_1_ enhancement, which is not observed in SPIONs.

### 3.4 *In vivo* biodistribution

**Fig. 5:**
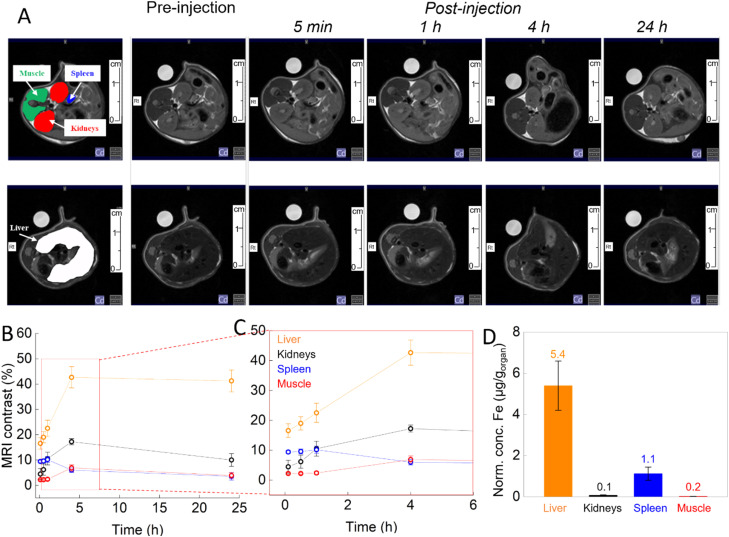
*In vivo* enhancement of the contrast of MRI and organ biodistribution over time after MND injection. (A) *T*_2_-weighted MRI images of the mouse abdomen acquired at 7 tesla at 5 minutes, 1 hour, 4 hours, and 24 hours after injection. Strong signal hypointensity is visible in the liver, indicating accumulation of MND. ROI analysis was performed in the liver, kidneys, spleen, and muscles as indicated. (B) Quantitative analysis of MRI contrast (%) over time for each organ. The liver shows the highest and most sustained contrast enhancement, peaking around 5 hours and persisting up to 24 hours. (C) The inset highlights early contrast changes (0–6 h), with the liver exhibiting the fastest uptake, followed by kidneys and spleen. Muscles show negligible enhancement throughout. (D) ICP-MS for quantification of ion in organs at 24 h after the i.v. of MNDs. Values are normalized to the iron concentrations measured in the corresponding organs of wild-type animals.

The *T*_2_-weighted MR images acquired at multiple time points after injection (5 minutes, 1 hour, 4 hours, and 24 hours) reveal a dynamic and organ-specific distribution of the contrast agent. As shown in Fig. 5A, a strong and sustained signal drop (indicative of contrast enhancement) was observed in the liver over time, consistent with accumulation of MNDs in the reticuloendothelial system.^67^ Quantitative analysis of MRI contrast enhancement over time is presented in the bottom panel. The liver exhibited the highest contrast enhancement, reaching approximately 45% at 4 hours and maintaining a similar level at 24 hours. In comparison, the kidneys showed moderate enhancement (around 15%), with a peak between 1 and 4 hours followed by a slight decrease. The spleen showed a slight transient increase, while muscle tissue remained nearly unchanged, indicating minimal MND accumulation in non-reticuloendothelial tissues. The inset graph in Fig. 5B highlights the early-phase kinetics of contrast uptake in the first 5 hours, confirming the faster and more pronounced uptake in the liver relative to other organs. These results support the preferential uptake of the contrast agent by liver tissue, likely mediated by phagocytic activity and hepatic clearance mechanisms. Confirmation of hepatic biodistribution was obtained by quantifying iron *via* ICP-MS, after subtraction of endogenous iron content (Fig. 5D). Analysis of the liver, kidneys, spleen, and muscle revealed a significantly higher iron uptake in the liver, with approximately 5.4 µg Fe^3+^ per g^−1^ of tissue. In contrast, the iron content in the other organs was markedly lower, measuring 1.12, 0.072, and 0.021 µg Fe^3+^ per g^−1^ for the kidneys, muscle, and spleen, respectively.

Although this study was performed on a small cohort of animals (*N* = 3), the high reproducibility of biodistribution following intravenous injection of MNDs is well established and primarily depends on the physicochemical properties of the particles, such as size, surface coating, charge, and hydrodynamic diameter. These parameters critically influence pharmacokinetics and cellular uptake, leading to consistent accumulation mainly in the reticuloendothelial system (liver, spleen) across animals under standardized conditions.^68,69^ Therefore, despite the limited sample size, the observed distribution patterns are expected to be representative. Nevertheless, further investigations involving larger cohorts will be required to confirm these observations, strengthen the statistical validity of the findings, and evaluate systemic delivery and long-term stability, which represent critical steps toward successful clinical translation and further development of this platform. From a methodological point-of-view, in this study, we monitored the longitudinal distribution of MNDs in mouse models using rapid *T*_2_*w* imaging. While *T*_2_*w* images are not inherently quantitative, this approach allowed us to follow temporal changes in contrast with minimal acquisition time, as commonly reported in the literature for dynamic assessment of contrast agents.^50^ Our results demonstrated that, despite the high *T*_2_ relaxivity of the MNDs, the accumulation in the liver was low, resulting in modest signal changes, in agreement with ICP-MS measurements.

Although this approach enabled effective longitudinal monitoring, the use of *T*_2_*w* imaging represents a limitation due to its qualitative nature. To overcome this, future studies will employ fully quantitative *T*_2_ mapping to assess organ-specific uptake of the particles. This will provide a more precise evaluation of particle distribution and kinetics, complementing the findings reported here.

## 4. Conclusions

Magnetic nanodiscs (MNDs) exhibit dual-mode relaxivity and strong potential as MRI contrast agents. Their high *r*_1_ relaxivity at ultralow fields (<0.1 T) and pronounced *r*_2_ enhancement above 3 T enable both *T*_1_ and *T*_2_ imaging applications. *Ex vivo* analyses confirmed bi-exponential *T*_2_ relaxation consistent with heterogeneous magnetic environments, while *in vivo* imaging revealed predominant hepatic uptake with delayed accumulation and transient renal and splenic distribution. In contrast to conventional iron-oxide systems, MNDs combine dual-mode relaxivity with vortex-mediated actuation capabilities, making them uniquely positioned for theranostic applications where MRI visibility and functional activation can be integrated. This study therefore establishes the relaxometric foundation necessary for the future use of MNDs as multimodal, activatable probes in neurotechnology and biomedical imaging.

## Author contributions

DG, GF, NT, AC: conceptualization, formal analysis, methodology, supervision, and writing. EK and EDG: data acquisition, analysis, and writing. SBN, VDT, LS, UA, TG, FW, RS, RT, CP, AC, MS and A C: data acquisition, analysis, and manuscript editing. All authors approved the final version of the manuscript.

## Conflicts of interest

There are no conflicts to declare.

## Abbreviations

CTRL: Control
DLS: Dynamic light scattering
DMEM: Dulbecco's modified eagle medium
DMSO: Dimethylsulfoxide
EMA: European Medicines Agency
FBS: Fetal bovine serum
FDA: U
GBCAs: Gadolinium-Based Contrast Agents
Hb: Hemoglobin
ICP-MS: Inductively coupled plasma mass spectrometry
IR: Inversion recovery, a type of MRI pulse sequence
LoD: Limit of detection
MNDs: Magnetite nanodiscs
MNPs: Magnetite nanoparticles
Magnetic resonance imaging: Magnetic resonance imaging
MSME: Multi-slice multi-echo, a type of magnetic resonance imaging scanning technique
MTT: Methylthiazolyldiphenyl-tetrazolium bromide, a chemical used in cell viability assays
NMR: Nuclear magnetic resonance
NMRD: Nuclear magnetic relaxation dispersion
NSF: Nephrogenic systemic fibrosis
PBS: Phosphate-buffered saline
PMAO: Poly(maleic anhydride-*alt*-1-octadecene)
RARE: Rapid acquisition with relaxation enhancement, a type of MRI scanning technique
RBC: Red blood cells
SAR: Specific absorption rate
SDS: Sodium dodecyl sulfate
SPIONs: Superparamagnetic iron oxide nanoparticles
ST%: Saturation transfer percentage, used in CEST imaging
*T*_1_ and *T*_2_: Longitudinal and transverse relaxation times, respectively
*T*_1_ *w* and *T*_2_*w*: *T*_1_-weighted and *T*_2_-weighted
TEM: Transmission electron microscopy
UP: Ultra-pure

## Supplementary Material

NA-OLF-D5NA01089F-s001

## Data Availability

All experimental data supporting this article, including magnetic measurements, relaxometry datasets, imaging files, and analysis code are available from the corresponding author upon reasonable request. All data comply with the Royal Society of Chemistry’s data sharing policies. There are no restrictions on data access. Supplementary information (SI): detailed descriptions of NMR relaxivity measurements, MRI acquisition protocols for phantom and *ex vivo* studies, data analysis procedures, and additional characterization data and figures. See DOI: https://doi.org/10.1039/d5na01089f.
