# Supplementary material for: Magnetite nanodiscs as vortex-enhanced MRI contrast agents: a novel approach in medical imaging
Source: Nanoscale Adv. 2026 Apr 1;8(9):2928–41. doi: 10.1039/d5na01089f (PMC13054993; doi:10.1039/d5na01089f)
Supplement: NA-008-D5NA01089F-s001 [file NA-008-D5NA01089F-s001.pdf]

Supplementary Information (SI) for Nanoscale Advances.
This journal is © The Royal Society of Chemistry 2026

Supplementary Information

Magnetite Nanodiscs as Vortex-Enhanced MRI Contrast Agents: A Novel Approach in Medical Imaging

Elif Koçar,[a]* Giuseppe Ferrauto[b]* Syed Bilal Nizami,[c] Vicente Durán Toro,[a] Uzair Ali,[a] Lorenzo Signorelli,[a] Teresa Giannattasio,[c] Marco Micali,[c] Franziska Wasner,[a] René Stein,[d] Marianna Sorrentino,[b] Alessia Corrado,[b] Chiara Papi,[b] Angelo Scarciglia,[b] Rainer Tietze,[d] Enza Di Gregorio,[b]**†, Nicola Toschi,[c,e]** Danijela Gregurec,[a]**† and Allegra Conti[c]**

Supplementary information on the Characterization of NMR Relaxivity (Experimental section, 2.5)

Variable-field T_1 values for different MNDs concentrations were measured using a Fast Field Cycling (FFC) relaxometer (Stelar, Italy) over the frequency range 0.001-20 MHz. Longitudinal relaxivities r_1 at each frequency were subsequently determined from the linear dependence of the relaxation rate ($1/T_1$) on the contrast agent concentration. Measurements were performed using pre-polarized or non-polarized pulse sequences depending on the magnetic field range, following the standard polarization-relaxation-detection field-cycling scheme [1]. At each selected magnetic field strength, the longitudinal magnetization recovery curve Mz(t) was acquired and independently analyzed. To extract the T1 value, tThe recovery curves were fitted using thea mono-exponential function:

$$M_z(t)=M_0(1-e^{-t/T_1}) \qquad (1)$$

where M_0 is initial transverse magnetization immediately after the excitation pulse and t is the time after the radiofrequency pulse. Data fitting was performed by least-squares regression using the manufacturer's analysis software (Stelar). Each point of the NMRD profile therefore represents an independent T_1 measurement obtained from time-domain fitting at a fixed magnetic field, without applying any global or field-dependent fitting model.

Longitudinal (T_1) and transverse (T_2) relaxation times were measured across the 20-80 MHz range using a Stelar relaxometer, with the magnetic field adjusted to match the proton Larmor frequencies at each measurement point. For each frequency, relaxation data were acquired as a function of the appropriate timing parameter, t, and analyzed independently.

T_1 values were obtained by fitting the magnetization recovery curves acquired with an inversion-recovery (IR) sequence to a mono-exponential model:

$$M_z(t)=M_0(1-2e^{-t/T_1}) \qquad (2)$$

T_2 values were obtained using a CPMG sequence by fitting the decay of the transverse magnetization (echo amplitudes) to a mono-exponential model:

$$M_{xy}(t)=M_{xy,0}\, e^{-t/T_2}) \qquad (3)$$

All fits were performed by least-squares regression using the manufacturer's analysis software (Stelar). Thus, each reported T_1 and T_2 value corresponds to an independent time-domain fit at a fixed magnetic field, without applying any global field-dependent model.

Detailed explanation of MR Acquisitions of Phantom and Ex Vivo Brains

T_2-weighted magnetic resonance acquisitions were performed on a custom-made agarose-based phantom designed to evaluate the transverse relaxivity (r_2) of MNDs. The phantom consisted of eight tubes embedded in a 1% agarose gel matrix, each filled with MND suspensions at different concentrations (100, 75, 50, 25, 10, 5, 2.5 µM, and deionized water as control). T_2 measurements were performed on dedicated agar-based phantoms using a multi-slice multi-echo (MSME) sequence on a 7 T Bruker MRI system (Bruker Avance 300 MHz spectrometer, equipped with microcoil). Two hundred echoes (TE = 3-600 ms, TR = 5000 ms) were acquired, and voxel-wise T_2 values were estimated by mono-exponential fitting of the signal decay.

To further investigate T_2-weighting, additional experiments were conducted using the same magnetic field (7 T, Bruker) with a multi-spin-echo sequence, varying the number of refocusing pulses (RF = 24, 32, and 64). Unlike standard multi-echo acquisitions at different TE values which alter T_2 weighting primarily by sampling signal decay at progressively later echo times, varying the number and configuration of refocusing pulses allows precise modulation of T_2-weighting independent of echo time, offering additional control over contrast generation and sensitivity to relaxation effects. This approach enables a more detailed assessment of how MNDs influence signal decay dynamics within the phantom, providing further insights into their behavior and improving sensitivity to concentration differences beyond what is achievable by simple TE variation alone. Representative *ex vivo* magnetic resonance images were obtained after stereotactic injection of 10 µL of MNDs (200 µM ([Fe]=1.04 mM) in a previously harvested mouse brain. T_2-contrast was assessed in images acquired through multi-spin multi-echo (MSME) MRI images (at 40 echo times (TEs) ranging from 10 to 400 ms with an increment of 10 ms, res (x,y,z) = 80, 100, 500 µm).

Quantitative T_2 maps were generated from MSME MRI data. For each voxel, signal decay was modelled using both mono-exponential and bi-exponential functions.

Mono-exponential model: $S(T_E)=S_0 \cdot e{-}T_E/T_2$

Bi-exponential model: $S(T_E)=A_1 \cdot e{-}T_E/T_{2,1}+A_2 \cdot e{-}T_E/T2,2$

Nonlinear least-squares curve fitting was performed using the Levenberg-Marquardt algorithm implemented via MATLAB's lsqcurvefit function.

To assess which model better described the observed signal decay, voxel-wise model comparison was performed using the Akaike Information Criterion (AIC) and the Bayesian Information Criterion (BIC). These criteria allow for quantitative model selection by balancing the goodness of fit with model complexity, penalizing overfitting associated with additional parameters.

The use of a bi-exponential model is motivated both by physical and experimental considerations. Physically, tissues with heterogeneous microenvironments, such as intra- and extracellular compartments, or regions with varying magnetic susceptibility can give rise to non-monoexponential decay behavior[2, 3]. The use of a bi-exponential model to fit T_2-weighted signal decay is particularly relevant in the context of local injections of magnetic particles. These particles introduce spatially varying magnetic field gradients, resulting in heterogeneous relaxation behavior within the affected region[4]. The model assumes the presence of two proton populations experiencing distinct magnetic

environments: one located closer to the injection site and the other farther away. Protons near the magnetic particles are subjected to stronger local field inhomogeneities, leading to faster transverse relaxation (shorter T_2), while protons located at a greater distance experience more homogeneous magnetic fields, resulting in slower relaxation (longer T_2). This dual-component relaxation reflects the microstructural alterations induced by the contrast agent and provides a more accurate representation of the signal decay dynamics in such complex environments[5].

By applying AIC and BIC, the fitting process allows for an objective, data-driven evaluation of whether the signal decay exhibits behavior consistent with a single or dual relaxation mechanism. In this framework, the bi-exponential model serves not only as a biophysically plausible alternative, but also as a tool to verify that the injected contrast agents produce the expected effects on local relaxation dynamics.

AIC and BIC metrics penalize model complexity, making them suitable for distinguishing between mono- and bi-compartmental behavior in T_2 decay:

$AIC=n\cdot\ln(nRSS)+2k;$

$BIC=n\cdot\ln(nRSS)+k\cdot\ln(n)$

where n is the number of data points (echoes), k is the number of parameters, and RSS is the residual sum of squares. The bi-exponential model was considered significantly better than the mono-exponential model for voxels where the difference in AIC or BIC exceeded 10 ($\Delta AIC > 10$ or $\Delta BIC > 10$). This threshold is commonly used in the literature on model selection to indicate strong evidence in favor of a more complex model[6, 7]. Binary voxel-wise maps were then generated to visualize the spatial distribution of regions where bi-exponential behavior predominated.

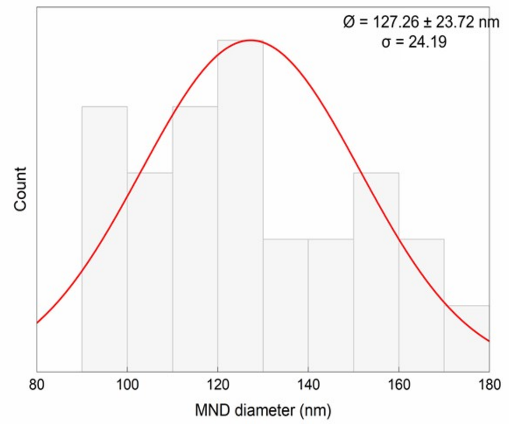

Figure S1: Size histogram for determining average MND diameter. Determined from 26 samples as the diameter across largest diagonal using ImageJ software

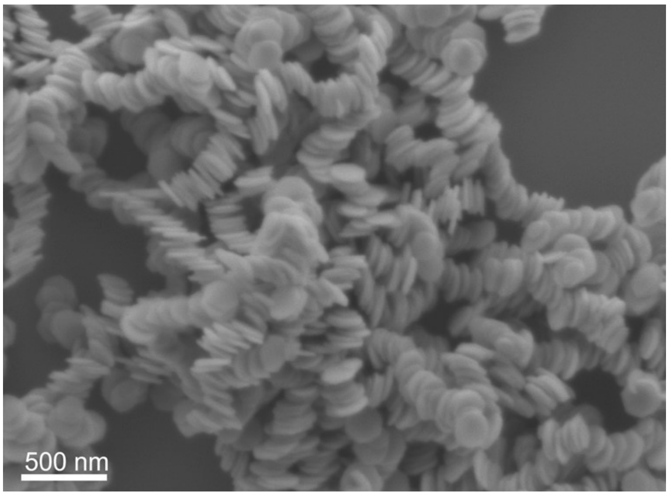

Figure S2: SEM micrograph of clustered MNDs upon large concentration deposition.

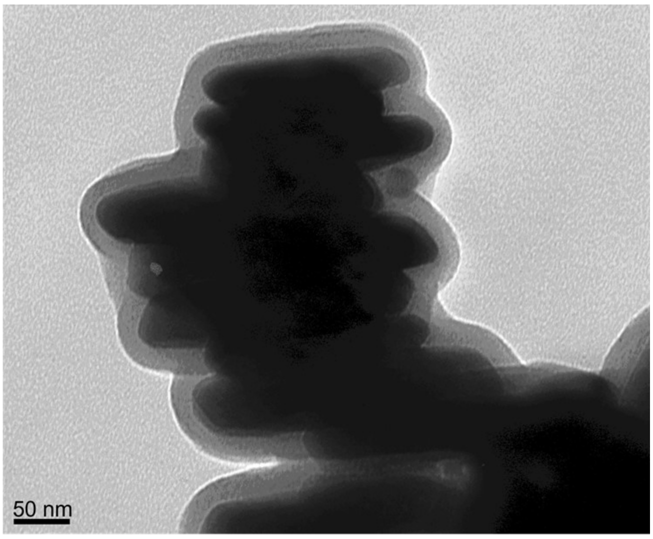

Figure S3: TEM micrograph of PMAO- coated MNDs.

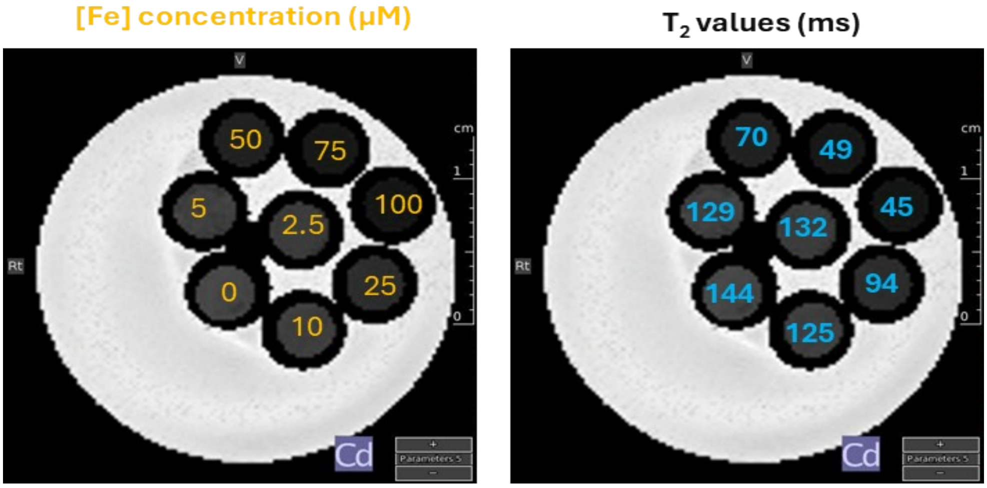

Figure S4: Left: Schematic of the phantom used for experiments. The phantom consisted of agarose tubes containing different MND concentrations (corresponding to iron concentrations from 2.5 to 100 μM, left). A T_2 map acquired at 7 T (right) was used to quantify the transverse relaxivity of the MNDs.

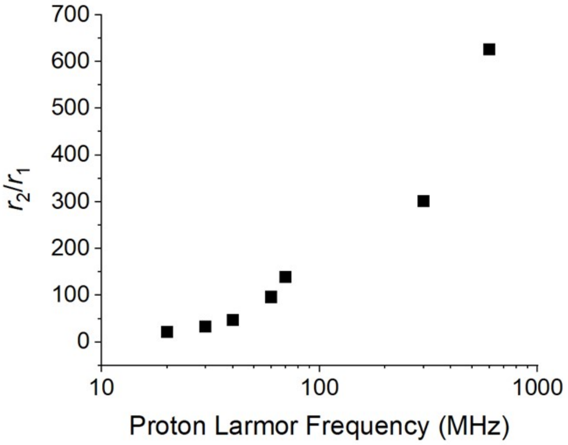

Figure S5. Transverse-to-longitudinal relaxivity ratios (r_2/r_1) of MNDs across the investigated magnetic field strengths (10-600 MHz). In our dataset, r_2/r_1 values range from approximately 25 to 650, with r_2 consistently much greater than r_1 at all field strengths. These high ratios indicate that the nanodiscs exhibit strong T_2 relaxivity dominance and behave predominantly as T_2 contrast agents. No field range showed r_2/r_1 values low enough to suggest significant T_1 contrast or dual-mode behavior.

REFERENCES

[1] R. Kimmich, E. Anoardo, Field-cycling NMR relaxometry, Progress in Nuclear Magnetic Resonance Spectroscopy 44(3) 257-320.

[2] W.C. Cole, A.D. Leblanc, S.G. Jhingran, The origin of biexponential T2 relaxation in muscle water, Magnetic Resonance in Medicine 29(1) (1993) 19-24.

[3] J. Duyn, MR susceptibility imaging, Journal of Magnetic Resonance 229 (2013) 198-207.

[4] R. Auccaise, Insights into NMR relaxation and susceptibility representation, Chemical Physics 591 (2025) 112583.

[5] A. Sharafi, G. Chang, R.R. Regatte, Biexponential T(2) relaxation estimation of human knee cartilage in vivo at 3T, J Magn Reson Imaging 47(3) (2018) 809-819.

[6] D.R.A. Kenneth P. Burnham, Model Selection and Multimodel Inference: A Practical Information-Theoretic Approach, Springer Science & Business Media (2002) 488.

[7] R.E. Kass, A.E. Raftery, Bayes Factors, Journal of the American Statistical Association 90(430) (1995) 773-795.
